# Multiple Traces of Families of Epoxy Derivatives as New Inhibitors of the Industrial Polymerization Reaction of Propylene

**DOI:** 10.3390/polym16142080

**Published:** 2024-07-21

**Authors:** Joaquin Hernandez Fernandez, Rodrigo Ortega-Toro, John R. Castro-Suarez

**Affiliations:** 1 Grupo de Investigation CECOPAT&A, Chemistry Program, Department of Natural and Exact Sciences, San Pablo Campus, University of Cartagena, Cartagena 130015, Colombia; 2 Chemical Engineering Program, School of Engineering, Universidad Tecnológica de Bolivar, Parque Industrial y Tecnológico Carlos Vélez Pombo Km 1 Vía Turbaco, Cartagena 130001, Colombia; 3 Department of Natural and Exact Science, Universidad de la Costa, Barranquilla 080002, Colombia; 4 Food Packaging and Shelf-Life Research Group (FP&SL), Food Engineering Department, Universidad de Cartagena, Cartagena 130015, Colombia; rortegap1@unicartagena.edu.co; 5 Área Básicas Exactas, Universidad del Sinú Seccional Cartagena, Cartagena 130015, Colombia; john.castro@unisinucartagena.edu.co

**Keywords:** polypropylene, catalytic efficiency, Ziegler–Natta system, inhibitors, impurities, polymerization, epoxides

## Abstract

In this study, the impact of ethylene oxide, propylene oxide, 1,2-butene oxide, and 1,2-pentene oxide on the polymerization of propylene at an industrial level was investigated, focusing on their influence on the catalytic efficiency and the properties of polypropylene (PP) without additives. The results show that concentrations between 0 and 1.24 ppm of these epoxides negatively affect the reaction’s productivity, the PP’s mechanical properties, the polymer’s fluidity index, and the PP’s thermal properties. Fourier transform infrared spectroscopy (FTIR) revealed bands for the Ti-O bond and the Cl-Ti-O-CH_2_ bonds at 430 to 475 cm^−1^ and 957 to 1037 cm^−1^, respectively, indicating the interaction between the epoxides and the Ziegler–Natta catalyst. The thermal degradation of PP in the presence of these epoxides showed a similar trend, varying in magnitude depending on the concentration of the inhibitor. Sample M7, with 0.021 ppm propylene oxide, exhibited significant mass loss at both 540 °C and 600 °C, suggesting that even small concentrations of this epoxide can markedly increase the thermal degradation of PP. This pattern is repeated in samples with 1,2-butene oxide and 1,2-pentene oxide. These results highlight the need to strictly control the presence of impurities in PP production to optimize both the final product’s quality and the polymerization process’s efficiency.

## 1. Introduction

Since their discovery in the 1950s, MgCl_2_-based Ziegler–Natta (ZN) catalysts have been used for most global polyolefin production. Due to its high commercial use, significant effort has been made to ensure constant progress in catalyst activity and stereospecificity [1].

The latest generation of ZN catalysts, requiring small amounts of titanium and aluminum alkyls, facilitates precise control of the morphology of the polymer particles and guarantees effective management of various aspects of the polymerization process [2]. However, the deactivation of ZN, which implies the progressive decrease in catalytic activity and selectivity, is a topic of great and constant interest in implementing the industrial catalytic process [3,4]. The deactivation mechanisms of ZN are diverse; however, they can be categorized into six intrinsic catalyst deterioration mechanisms: (1) poisoning, (2) clogging, (3) thermal degradation, (4) formation and release of compounds in vapor accompanied by migration from the catalyst surface or particle, (5) vapor–solid and solid–solid interactions, and (6) wear/crushing [5]. When considering these processes, the reasons for deactivation fall mainly into three categories: chemical, physical, and thermal. Mechanisms 1, 4, and 5 belong to the chemical field, while 2 and 6 are physical [6].

Within deactivation processes, poisoning is an important aspect that is not yet fully understood. This phenomenon is characterized by the strong chemical binding of impurities at sites that would otherwise be available for catalytic function. Thus, poisoning has practical implications. Whether a substance functions as a poison depends on its adsorption capacity to other substances competing for catalytic sites. ZN inhibitors deplete both the catalyst and the co-catalyst, leading to an increase in operating costs and the remaining concentration of the catalysts in the final product [6].

In the context of ZN catalysts that use titanium (TiCl_4_/MgCl_2_), they are particularly susceptible to certain organic compounds that can function as poisons, decreasing the efficiency of polymer production. Generally, these compounds mainly comprise carbon oxides [7], alcohols [8], mercaptans [9,10], water, and oxygen, among others [11,12,13]. It is widely known that organoaluminum compounds react rapidly with the most common catalytic contaminants, transforming them into other compounds, typically aluminum alkoxides R_2_AlOR′ [14], which have a less negative impact on the catalyst’s performance. Other poisons act more aggressively, stopping the polymerization reaction [15]. Including a toxic molecule in the catalytic system can affect several aspects of the catalysis process. It can interact with the MgCl_2_ surface, the Ti active center, the catalytic active site environment, and even coordinate with the co-catalyst (TEAL), impacting activity and stereospecific selectivity [16].

This study aims to investigate the potential interactions between poisonous molecules, such as epoxides, during the polymerization process of polypropylene. The impact of these interactions on the mechanical properties and thermal degradation of the resulting material will be analyzed by incorporating different concentrations of four types of epoxides (ethylene epoxide, propylene, 1,2-butene, and 1,2-pentene). No comprehensive study has been conducted on how these impurities affect catalyst sensitivity and productivity in this context, underscoring the importance and relevance of research such as the present one. The study will include the intentional injection of various concentrations of these poisons at different stages of the polymerization process. The amount of polymer produced in the presence and absence of poisons will be evaluated, and the presence of these substances will be investigated to determine how the presence of these substances affects the productivity of the catalyst. In addition, the produced polymer will be characterized using techniques such as infrared spectroscopy and thermogravimetric analysis (TGA), and the melt flow index (MFI) will be measured to understand the material’s properties better.

## 2. Materials and Methods

### 2.1. Reagents

Oxides of ethylene, propylene, 1,2-butene, and 1,2-pentene purchased with different degrees of purity (≥99.5%, ≥99%, 99%, and ≥98.0%) were used from MERK in Darmstadt, Germany, supplied by Avantor via VWR. Using an online diffusion system, these compounds were combined with residual liquefied petroleum gas (LPG) at varying concentrations between 0.09 and 6 ppm. The polymerization of propylene was carried out under meticulously designed experimental conditions. A fourth-generation Ziegler–Natta catalyst was used, supported mainly in magnesium chloride (MgCl_2_). As the internal donor agent, di-isobutyl phthalate (DIBP) from Sudchemie was used. Additionally, cyclohexylmethyl methyldimethoxysilane (CMDS) from MERK was incorporated as an external donor agent. High-purity tri-ethylaluminum (TEAL), obtained from MERK and diluted in n-heptane, activated the catalyst and maintained the system’s stability. This study used high-purity polymer-grade propylene supplied by Shazand Petrochemical (Arak, Iran) as the primary raw material.

### 2.2. Inhibitor Injection

**Figure 1:**
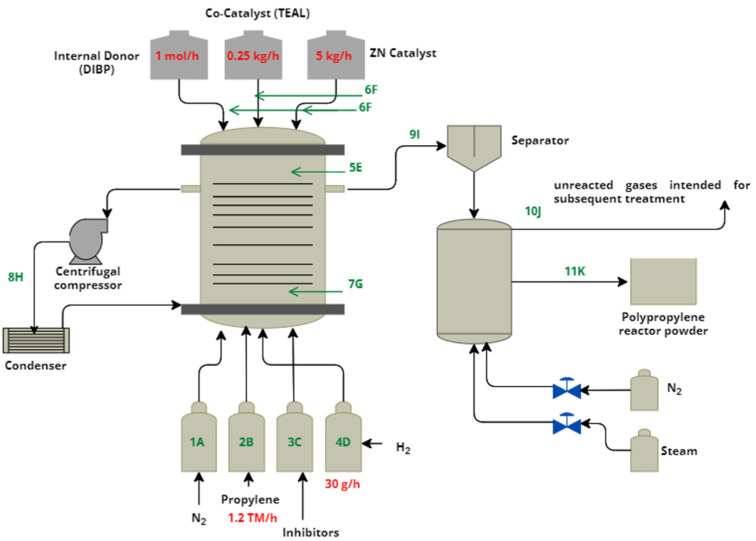
Polymerization Process and Injection Points of the epoxides.

The gas phase polymerization was carried out meticulously using the ZN catalyst, following the method proposed by Hernández [17]. The process was developed in a fluidized bed reactor previously purged with nitrogen to ensure ideal reaction conditions. The feed included 30 g of hydrogen per hour (30 g/h) and 1.2 metric tons of propylene per hour (1.2 TM/h) introduced from the base of the reactor. In addition, 0.25 kg per hour of TEAL (0.25 kg/h), 1 mol per hour of selectivity control agent (1 mol/h), and 5 kg per hour of ZN (5 kg/h) were added. Once the reaction was completed, acetone was added to stop the polymerization, transferring the resulting suspension to a receiving flask in a nitrogen atmosphere (1 atm N_2_), as illustrated in Figure 1.

The optimal reaction conditions were maintained in this batch-type operation at a temperature of 70 °C and pressure of 27 bar. The monomer supply line received precise additions of 1,2-butene, 1,2-pentene, propylene oxides, and ethylene, described in detail in Table 1.

### 2.3. Melt Flow Index (MFI)

A Tinius Olsen (Red Hills, UK) MP1200 plastometer was used to determine the melt flow index (MFI). The operating temperature in the cylinder of the equipment was maintained at 230 °C, using a 2.16 kg piston to displace the molten material.

### 2.4. Evaluation of the Mechanical Properties of the Polymer

The mechanical properties of the polymer were evaluated by processing thermoplastic composites in a twin-screw extruder, followed by injection molding. The test specimens were manufactured according to ASTM standards, using specific molds to perform tensile (ASTM D638, https://www.astm.org/d0638-14.html, accessed on 30 June 2024) and flexural (ASTM D790, https://www.astm.org/d0790-17.html, accessed on 30 June 2024) tests. The mold temperature was maintained at 50 °C. The tensile test was carried out using an H50KL universal testing machine (Tinius Olsen), following ASTM D638 standards. During the tensile test, a uniaxial load was applied to the ends of the specimen. Once fixed in the jaws, the electromechanical system stretched the material vertically. The injection molded specimens were classified as TYPE I, with a gauge length (G) of 50 mm, a narrow section width (W) of 12.7 mm, and a thickness (T) of 3.4 mm.

The flexural strength was evaluated using the H50KL equipment (Tinius Olsen) according to the ASTM D790 standard. The Izod impact test was carried out with an impact pendulum model IT504 (Tinius Olsen) in accordance with the ASTM D256 (https://www.astm.org/d0256-23e01.html, accessed on 30 June 2024) standard. For this test, one end of the specimen was clamped with a cantilever vise, and a 45° AV notch with a depth of 2.5 mm was created.

### 2.5. Rheological Measurements

In the cone-plate configuration, rheological measurements were carried out using a Carri-Med CSL-50 strain-controlled rheometer (Carri-Med Instruments Ltd., Dorking, UK). Cones with diameters of 40 mm and an angle of 4 degrees were used, as well as 60 mm cones with an angle of 1 degree. Small amplitude oscillatory shear tests were performed at a frequency range between 0.1 and 10 Hz to ensure they remained within the linear viscoelastic domain. Special attention was paid to reducing the applied stress and avoiding sample disturbances during frequency sweeps.

### 2.6. Thermogravimetric Analysis (TGA)

The thermal degradation analysis of the samples was carried out using a TGA Q500 thermogravimetric analyzer from TA Instruments, New Castle, DE, USA. The samples were subjected to a heating protocol with a temperature increase rate of 10 °C per minute, ranging from 40 °C to 800 °C. The analysis was conducted in an air environment with a constant flow of 50 cm^3^ per minute.

### 2.7. Infrared (IR) Spectroscopy

Infrared (IR) spectroscopic studies were conducted using a Nicolet iN10MX spectroscope from Thermo Fisher Scientific (Thermo Scientific, Waltham, MA, USA) equipped with an iN10Z unit. Attenuated total reflection (ATR) mode was used. Spectra were recorded with a resolution of 4 cm^−1^, spanning a range from 400 to 4000 cm^−1^, allowing sensitive and accurate identification of various absorption bands.

## 3. Results

### 3.1. Reaction of REAL with Epoxies

**Figure 2:**
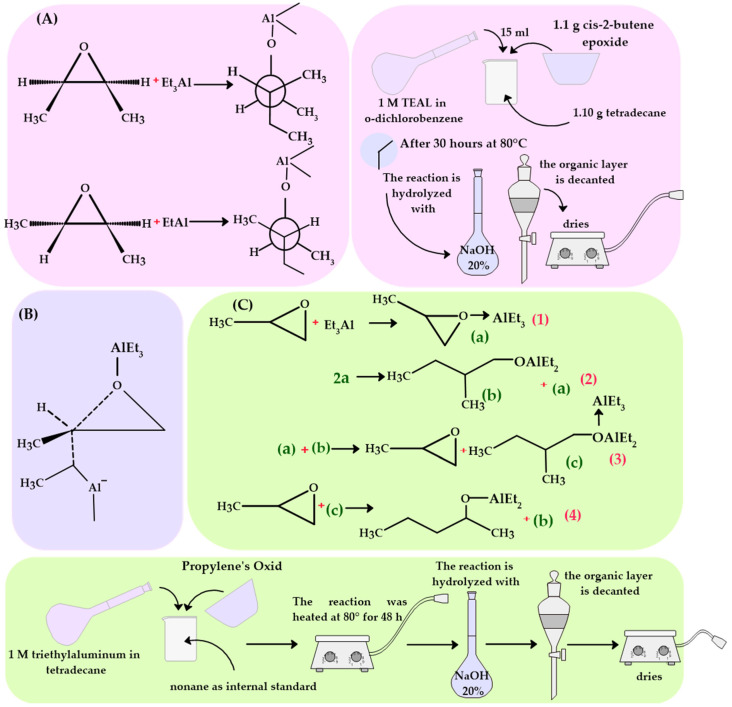
(**A**) Reaction of cis- and trans-2,3-epoxybutanes with tri-ethylaluminum; (**B**) Breakage of the secondary oxygen-carbon bond during the formation of the new bond with the attacking group; (**C**) Reaction between tri-ethylaluminum and propylene oxide.

Tri-alkylalanes are very effective Lewis acids that form complexes and reactions with various oxygenates. Very few studies have been conducted on the multiple aspects of the responses between tri-ethylaluminum and epoxides. However, the initial reaction stage between tri-ethylaluminum and epoxides is believed to involve coordination to form a complex through a bond. The diffusion of oxygen to the aluminum atom is similar to what happens with ethers and carbonyl compounds [18]. The reaction of cis- and trans-2,3-epoxybutanes with tri-ethylaluminum involves a predominant stereospecific opening of the epoxide ring at the back (Figure 2A). The ethyl groups attached to aluminum that are in complex with the reactant epoxide molecule are not in a steric position conducive to attacking the epoxide from the opposite side of the ring. This suggests that the reaction must be at least bimolecular; That is, the attack must arise from a tri-ethylaluminum molecule (free or in complex) that is not directly associated with the epoxide that is being affected.

The reaction between tri-ethylaluminum and propylene oxide under specific conditions results in a predominant opening of the ring at the back, especially notable with cis and trans epoxybutanes, as we observed in the previous case. This process mainly generates 2-methyl-1-butanol as the main product. The proposed mechanism for this reaction must reconcile the observed stereospecificity, which implies a ring opening following an SN_2_-type mechanism, suitable to explain the direction of the ring opening in propylene oxide. This type of SN_2_-modulated mechanism finds similarities in similar epoxide reactions, highlighting the importance of understanding how specific conditions and stereochemistry affect the reaction outcome.

In the reaction of epoxides with tri-ethylaluminum, a formation of products and stereochemistry similar to the well-known acid opening of epoxides is observed. It is postulated that a significant cleavage of the secondary oxygen-carbon bond occurs during the forming of the new bond with the attacking group. This expectation is based on the nature of allane, which acts as a weak nucleophile. In the case of using toluene as a solvent, it is observed that it tends to prefer the attack of the complex formed by allane and epoxide over tri-ethylaluminum [18]. Although the specific stereochemistry of propylene oxide opening has not yet been determined, the process is expected to occur with inversion, as does the opening of analogous 2,3-epoxybutanes (Figure 2B).

When the molar ratio is unity or less, the interaction between tri-ethylaluminum and propylene oxide follows two different mechanistic trajectories, the importance of which depends on the proportion of tri-ethylaluminum in relation to propylene oxide. This phenomenon is similar to what Ashby and his colleagues observed in the reaction of tri-methylaluminum with benzophenone [19]. The reaction process between tri-ethylaluminum and propylene oxide could include the steps described in Equations (1)–(4) (Figure 2C).

After analyzing the interaction between tri-ethylaluminum and propylene oxide, it is deduced that this interaction can inhibit the process and poison the catalyst in several ways. The complex formed between the epoxide and TEAL can block the active sites of the Ziegler–Natta catalyst. This blockage occurs because the epoxide, when coordinating with TEAL, can form species that are not as effective for the polymerization of propylene. This alters the structure or ability of the catalyst to interact with propylene optimally, therefore reducing catalytic efficiency and affecting polypropylene production.

### 3.2. Evaluation of the Impact of Different Oxides on the Reduction of Catalytic Productivity Depending on Their Concentration

**Figure 3:**
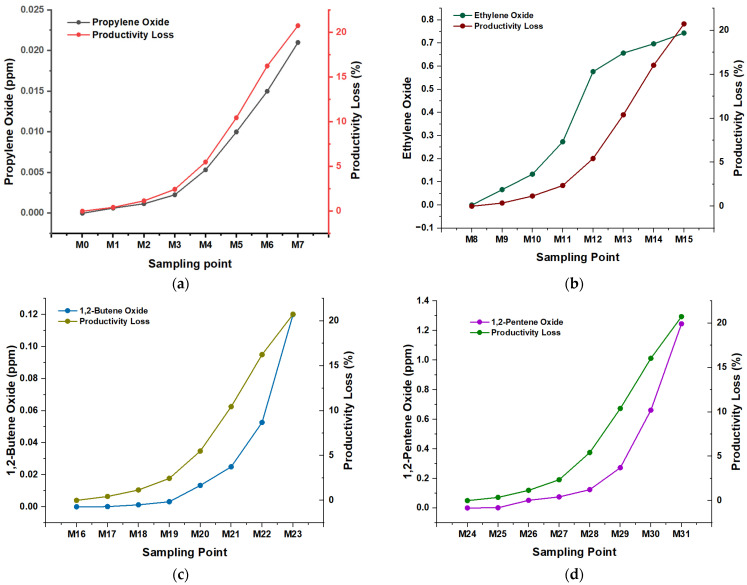
Loss of productivity (%), depending on the inhibitor (ppm) and type of sample; (**a**) Propylene oxide; (**b**) Ethylene oxide; (**c**) butene 1,2-oxide; (**d**) 1,2-pentene oxide.

The variability in the concentrations required of different inhibitors to induce a loss of catalytic productivity of 20% in the ZN catalyst suggests significant differences in the molecular interaction and interference capacity of each compound (Figure 3). This observation is based on several structural and kinetic considerations that influence the effectiveness of the inhibitors during the polymerization process.

First, each inhibitor’s molecular structure plays a crucial role. The geometry and chemical functionality determine the affinity and binding capacity of the inhibitor with the active site of the catalyst. For example, propylene oxide, with a structure prone to form specific interactions with ZN, shows greater efficiency at relatively low concentrations, such as 0.021 ppm, inducing a loss of 20% (Figure 3a). In contrast, 1,2-pentene oxide requires a concentration of 1.24 ppm to produce the same effect, indicating a lower affinity or a less efficient interaction with the catalyst (Figure 3d). The solubility and mobility of the inhibitors in the reaction matrix are also determining factors. More soluble and mobile inhibitors can reach the catalyst’s active sites more efficiently, which could explain why lower concentrations are effective for some compounds. More soluble inhibitors can diffuse more effectively through the reaction medium, allowing them to rapidly interact with the catalyst and cause changes in its activity. For example, with favorable solubility, ethylene oxide shows high effectiveness at relatively low concentrations, such as 0.74 ppm in sample M15 (Figure 3b), inducing a 20% loss in catalytic productivity. In contrast, less soluble inhibitors may require higher concentrations to achieve comparable effects. This is reflected in the case of 1,2-pentene oxide, which requires a concentration of 1.24 ppm to produce a 20% loss, suggesting lower mobility and a limited ability to disperse in the reaction medium and efficiently reach the catalyst active sites.

In addition to solubility, the mobility of inhibitors within the polymerization system plays a crucial role. The ability of an inhibitor to move freely in the reaction medium directly affects its probability of interacting with the catalyst. Inhibitors with high mobility can diffuse rapidly to the catalyst’s active sites, increasing their effectiveness at lower concentrations. This behavior is observed in propylene oxide, which shows high effectiveness at concentrations of 0.021 ppm in sample M7, causing a 20% loss in the productivity of the ZN catalyst. In addition to structural properties, specific chemical interactions play a critical role. Some inhibitors can establish specific bonds or non-covalent interactions with functional groups of the catalyst, increasing their ability to interfere with catalytic activity. This phenomenon is observed in the case of 1,2-butene oxide, where concentrations of 0.12 ppm in the M23 samples are sufficient to induce a loss of 20% (Figure 3c), highlighting a particularly effective chemical interaction.

### 3.3. Evaluation of the Influence of Oxide Concentrations on Polymer Properties and Polypropylene Production

#### 3.3.1. Impact of Inhibitors on the Bending Modulus and Production of Polypropylene

**Figure 4:**
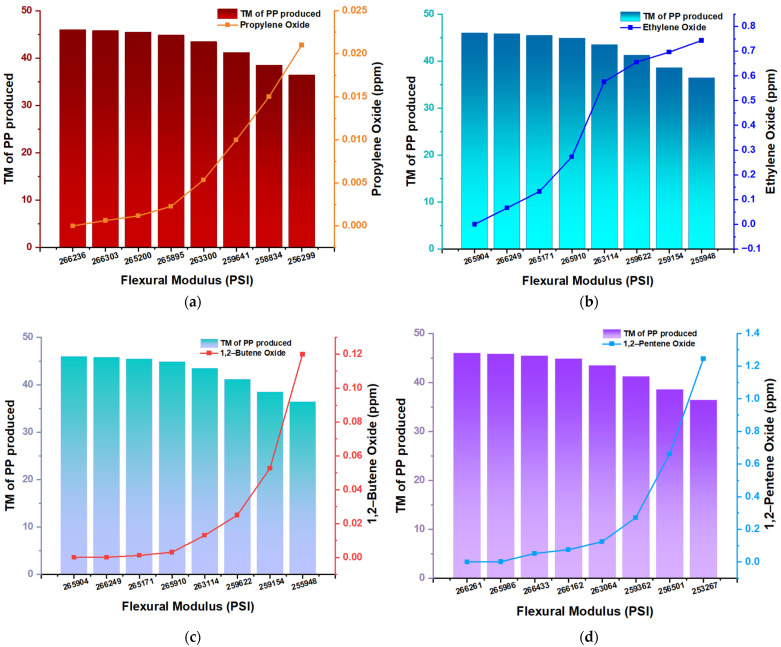
Flexural Modulus (PSI) and Productivity of PP (TM) as a function of the concentration of inhibitors in traces (ppm); (**a**) propylene oxide; (**b**) ethylene oxide; (**c**) butene 1,2–oxide; (**d**) 1,2–pentene oxide.

The flexural strength of polymeric materials defines the maximum bending stress the material can withstand when under flexural loading conditions. This characteristic is crucial to evaluate the flexural behavior of polymeric composites, which is essential for structural applications [20]. In this study, the impact of different concentrations of inhibitors (oxides) on the properties of PP has been evaluated, specifically on the flexural modulus and total yield. The results indicate that the presence of these inhibitors, even at deficient concentrations, significantly affects both the rigidity of the polymer and the quantity produced (Figure 4).

In the absence of propylene oxide (0 ppm), the flexural modulus of PP is 266,236 PSI, with a yield of 45 metric tons (TM). With the increase of the inhibitor concentration to 0.021 ppm, the flexural modulus decreases to 256,299.6 PSI, and the production drops to 35 TM (Figure 4a). This behavior suggests an inverse relationship between the concentration of the inhibitor and the properties of the PP, indicating that the presence of propylene oxide weakens the polymer structure and reduces production. Similarly, at 0 ppm ethylene oxide, the flexural modulus is 265,904 PSI, producing 45 TM of PP. Increasing the inhibitor concentration to 0.75 ppm, the flexural modulus drops to 255,948.3 PSI, and the production reduces to 35 TM (Figure 4b). For butene 1,2-oxide, a similar trend is observed. Without an inhibitor (0 ppm), the flexural modulus is 265,904 PSI, and the production is 45 TM. At a concentration of 0.12 ppm, the flexural modulus decreases to 255,948.3 PSI and the output to 35 TM (Figure 4c). Analysis of pentene 1,2-oxide reveals that the flexural modulus in the absence of the inhibitor is 266,261.3 PSI with a yield of 45 TM. Increasing the concentration to 1.24 ppm reduces the flexural modulus to 253,267.6 PSI, and the production drops to 35 TM (Figure 4d). Although a higher concentration of this inhibitor is required to observe significant deterioration, the negative impact on PP properties is clear. This pattern highlights the sensitivity of PP to the presence of different oxides, compromising both the rigidity of the material and the amount of product obtained.

#### 3.3.2. Impact of Inhibitors on the Flow Index and Molecular Weight of Polypropylene

**Figure 5:**
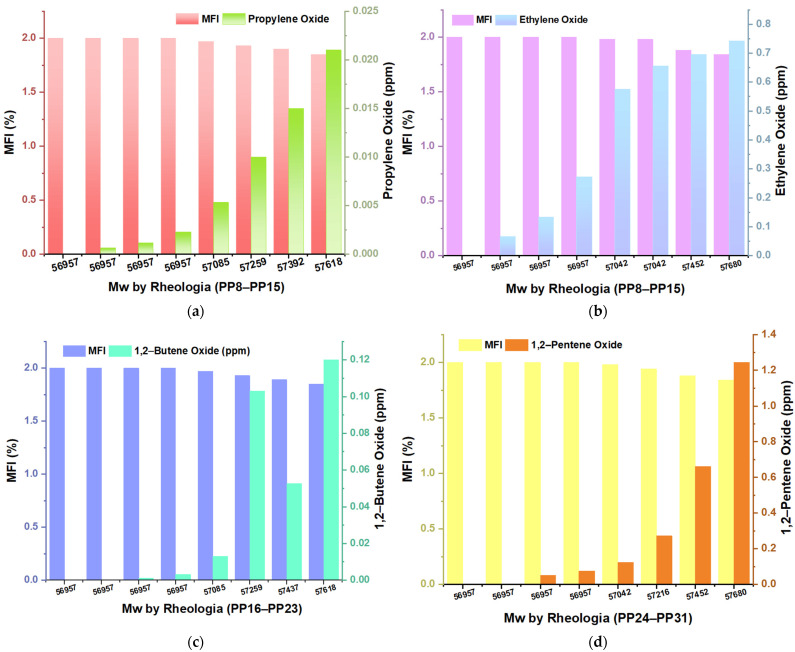
Average Molecular Weight (Mw), Melt Fluidity Index (MFI) as a function of the concentration of Inhibitors: (**a**) propylene oxide; (**b**) ethylene oxide; (**c**) butene 1,2-oxide; (**d**) pentene oxide.

In this study, the effect of different concentrations of the oxides on the properties of PP was investigated, focusing on the melt flow index (MFI) and the average molecular weight (Mw) (Figure 5). When analyzing the graphs corresponding to the different oxides, an increasing trend of the MFI of PP is observed, inversely proportional to the concentration of the inhibitors. The results show that the highest MFI was obtained when the concentration of inhibitors was minimum (0 ppm), indicating better fluidity of the polymer under these conditions. On the contrary, as the concentration of inhibitors increases, as observed with 0.021 ppm for propylene oxide (Figure 5a) and 0.74 ppm for ethylene oxide (Figure 5b), a significant decrease in the MFI of the PP. Regarding the Mw, stability is observed from sampling point M0 to M3, followed by notable variations from sampling point M4. During this period, a gradual but minimal increase in the Mw of PP is identified, coinciding with a proportional increase in the concentrations of the inhibitors.

The observation that higher concentrations of inhibitors correlate with a reduction in the MFI of PP indicates a direct interference in the polymer’s processability. This effect can be attributed to the formation of stable complexes between the inhibitors and monomers during the polymerization reaction, which affects the polymer’s molecular mobility and flow efficiency. On the other hand, the gradual increase in the Mw of PP with increasing concentrations of inhibitors suggests a possible modification in the length of the polymer chains. This could be due to the structural presence of inhibitors in the polymer matrix, which can act as starting or stopping points in the polymerization chain, thus affecting the final molecular mass of the polymer.

### 3.4. Thermal Degradation of the Polymer Due to the Presence of Different Oxides

**Figure 6:**
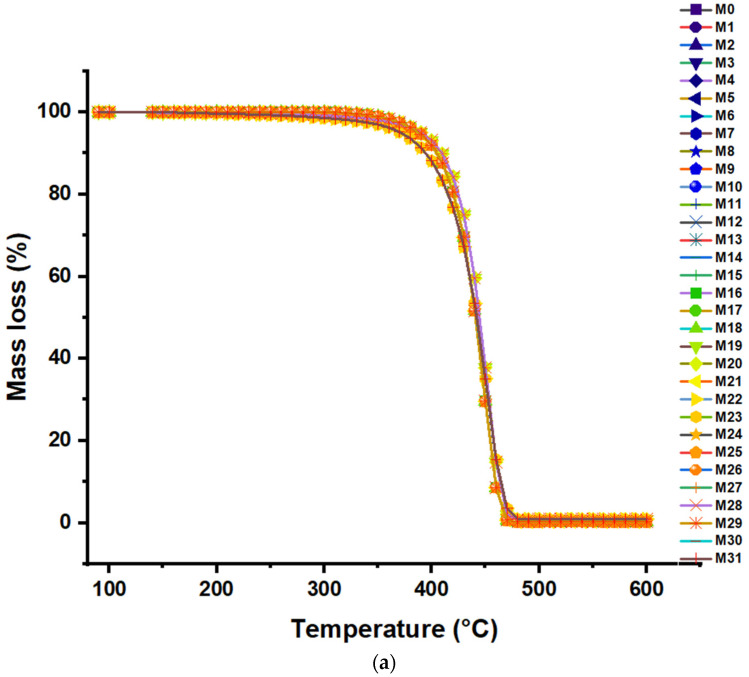

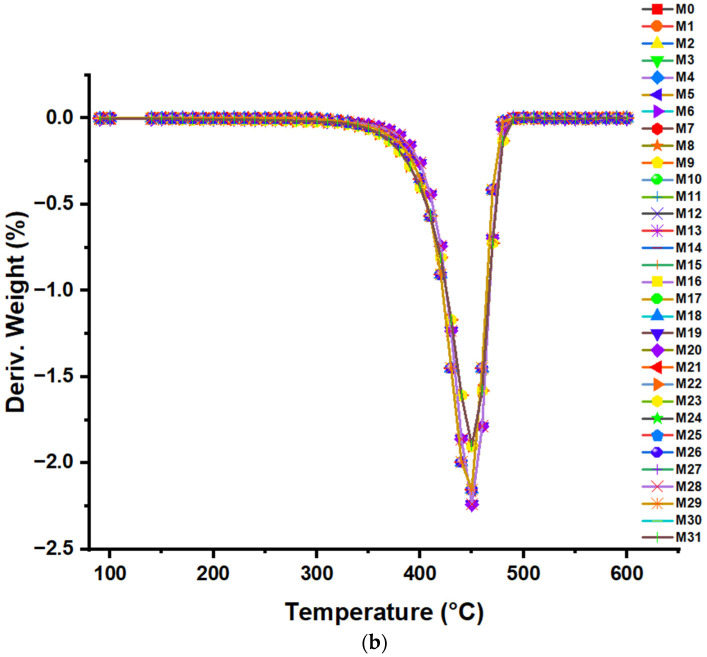
(**a**) Thermogravimetric degradation graph (TGA); (**b**) Derived thermogravimetric analysis (dTGA).

The thermal decomposition of the resins obtained with different concentrations of the four oxides was analyzed using thermogravimetric equipment (TGA, Figure 6). Thermal stability and decomposition behavior were evaluated in the first seven samples labeled M0–M7. The mass loss (%), as a function of temperature (°C), is recorded in Figure 6a. The TGA analysis of samples M0 to M7 reveals a highly similar thermal behavior. All samples maintain thermal stability up to approximately 300 °C, from which point a rapid and pronounced decomposition begins that extends up to around 450 °C. This inflection point is crucial to determine each sample’s decomposition onset temperature (Ti). However, since the differences are minimal, we can infer that the Ti is practically the same for all samples (M0–M31). The rapid mass loss observed may indicate the volatilization and decomposition of thermolabile components present in each sample. Since the decomposition region is consistent along all curves, it implies a similar composition between the samples. Subsequently, the mass loss curves stabilize, reaching a constant residual mass of 600 °C. This residual mass indicates the presence of non-volatile materials or carbonaceous residues that do not decompose at the analyzed temperatures. The similarity in the residual masses can indicate a consistency in the composition of the inorganic or non-volatile components of the samples.

The derived thermogravimetric analysis (dTGA) plot shows that all samples (M0 to M7) exhibit similar thermal behavior, with a notable weight loss occurring predominantly between 400 °C and 500 °C, indicating this temperature range as the main decomposition interval (Figure 6b). The thermal stability of the samples is maintained up to 400 °C, at which time a significant drop in the weight derivative is observed. Then, the curves stabilize again after 500 °C. However, sample M7, with the highest concentration of propylene oxide (0.021 ppm), shows a slight difference in its decomposition profile, suggesting that a higher inhibitor concentration can influence the thermal decomposition of the material. From sample M8 to M31, the exact behavior of samples M0–M7 can be observed.

### 3.5. FTIR Results

**Figure 7:**
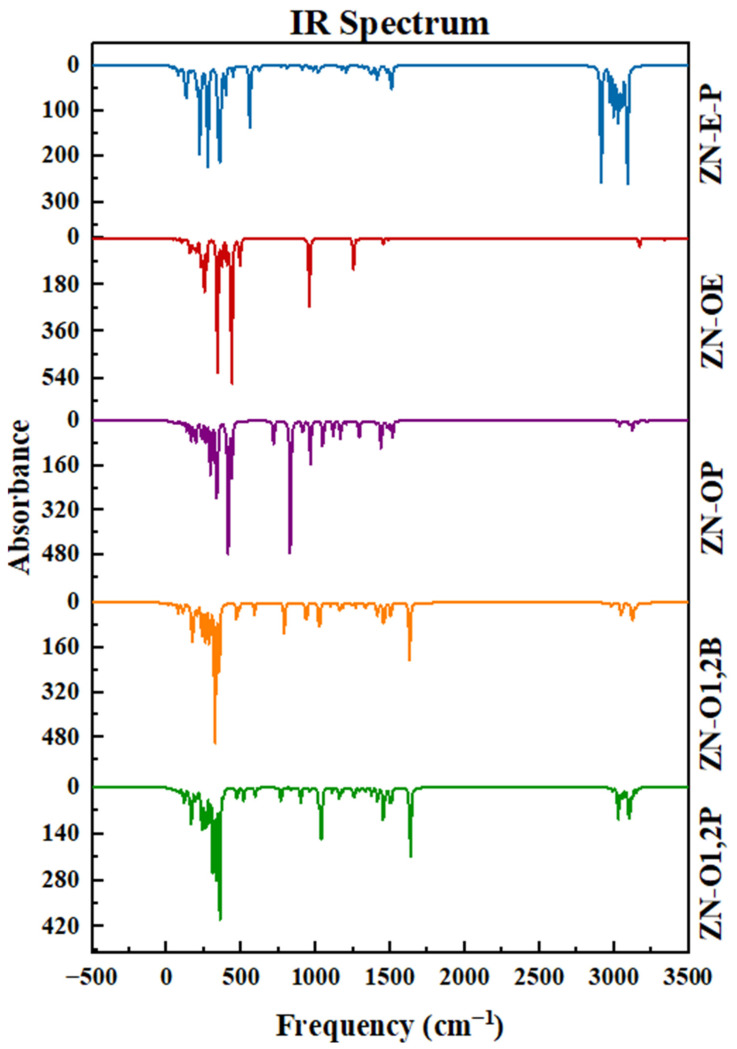
ZN-Ethylene-propylene (ZN-E-P) IR spectra; ZN-Ethylene Oxide (ZN-OE); ZN-Propylene oxide (ZN-OP); ZN-1,2-Butylene Oxide (ZN-O1,2B); ZN-1,2-pentene oxide (ZN-O1,2P).

The joint interpretation of the Fourier transform infrared (FTIR) spectra of the oxides of propylene, ethylene, 1,2-butene, and 1,2-pentene in the presence of the Ziegler–Natta catalyst offers a detailed view of their interaction with the active center of titanium. FTIR spectra reveal significant changes in the structure and chemical bonds of the oxides when in contact with the catalyst, providing a deeper understanding of the catalysis processes and surface dynamics in Ziegler–Natta systems. The graphic representation of the infrared (IR) spectra obtained from the complexes formed between the various oxides and the Ziegler–Natta catalyst is presented in Figure 7. In the upper part of the graph, the spectrum of the catalyst bound to ethylene is observed, mediated by the co-catalyst in the polymerization process, together with its respective monomer, propylene. The Ti-Cl bond is detected in the region from 618 to 555 cm^−1^ for the ZN-E-P complex. This finding, although it does not coincide precisely with the frequency range indicated by Eldin Redzic [21], suggests the formation of complexes or the adsorption of species on the catalytic surface, where the Ti-Cl bond is identified in the range of 603 at 617 cm^−1^, showing a closeness in the region of this bond.

Additionally, a peak was observed in the frequency range of 1510 cm^−1^, characteristic of the Cl-Mg bond present in the catalyst support. The presence of peaks between 1474 cm^−1^ and 1510 cm^−1^ corresponds to the stretching vibration of the C-H bond, particularly associated with the -CH group, followed by the -CH_3_ group, whose values are 2972 at 2990 cm^−1^. These results are consistent with Morteza Abazari et al. [22].

The second spectrum corresponds to the catalyst linked to the ethylene oxide inhibitor (ZN-OE). In this spectrum, peaks starting at 430 cm^−1^, when compared with the existing literature, reveal a similarity in the frequency range for the characteristic vibration of the Ti-O bond. Subsequently, a shift of the peak to the left is observed for the Ti-Cl bond, appearing at 493 cm^−1^, in contrast to propylene oxide, where the shift is directed to the right, reaching a frequency value of 725 cm^−1^. This phenomenon can be attributed to the adsorption effect of the inhibitors on the catalytic surface, as for the -CH_2_ group in the ZN-OE spectrum, a frequency range spanning from 1260 to 1456 cm^−1^ is detected.

Furthermore, the peaks corresponding to the Ti-O bond for the ZN-OP, ZN-O1,2B, and ZN-O1,2P spectra showed very similar characteristics with minimal shifts, which can be attributed to the different structural conformations of the inhibitors by binding to the catalytic surface. These spectra present vibrations at 440 cm^−1^, 475 cm^−1^, and 475 cm^−1^, respectively (see Table 2).

Regarding the Ti-Cl bond, a variation in the frequency of appearance of the peak was observed in the ZN-OP spectrum, with a pronounced peak at 725 cm^−1^. In contrast, the ZN-OE spectrum’s peak appears at 493 cm^−1^, indicating a significant discrepancy. For the ZN-O1,2B and ZN-O1,2P spectra, the binding frequencies for Ti-Cl remained similar, with values of 591 cm^−1^ and 599 cm^−1^, respectively. In the spectra corresponding to the epoxides, specifically ZN-OE, ZN-OP, ZN-O1,2B, and ZN-O1,2P, a peak of crucial importance was identified that confirms the reactions indicated in Table 3, corresponding to the Cl-Ti-O-CH_2_ bond, by the literature. The values reported for this type of vibration correspond to 718, 845, and 1006 cm^−1^.

## 4. Discussion

This research studies the influence of oxides on polypropylene polymerization, emphasizing their role in modulating the catalytic activity and the resulting properties of the polymer. Previous studies on the adsorption o impurities in catalytic systems and their impact on catalytic activity are fundamental to understanding these effects.

The results show that the thermal degradation of PP in the presence of epoxides such as propylene oxide, ethylene oxide, butene 1,2-oxide, and pentene 1,2-oxide follows a similar trend but varies in magnitude depending on the concentration of the inhibitor. For example, sample M7, containing 0.021 ppm propylene oxide, exhibits significant mass loss at 540 °C and 600 °C, suggesting that even small concentrations of this epoxide can markedly increase the degradation of thermal polypropylene. This behavior is consistent with the observations of Naeimeh Bahri-Laleh [6], who showed that poisons firmly stabilize the crystalline surfaces and the active centers of the catalyst, which could explain the more significant degradation observed due to the interaction of the epoxides with the PP. The analysis of the graphs shows an increasing trend in the MFI with increasing concentrations of epoxides, which is inverse to the behavior of the concentrations of oxides. This phenomenon is manifested when the concentration of the inhibitors increases (for example, 0.021 ppm for propylene oxide and 0.74 ppm for ethylene oxide), resulting in a decrease in the MFI of the polymer. This indicates that epoxides have a significant impact on the melt flow rate of polypropylene, possibly due to the formation of stable complexes during the polymerization reaction, which alters the structure of the polymer chain and, therefore, its final properties as the Mw. These findings align with the work of Daniel Christian Pernusch [23], who highlighted how impurities can affect the catalytic activity and molecular weight distribution in Ziegler–Natta catalyst systems. His findings indicated that when the concentration of poisons for the catalyst exceeds 120 ppm, the catalytic system’s activity significantly decreases. Molecular weight distribution (MWD) deconvolution analysis revealed that poisons (pyridine and n-butanol) affect the composition of the resulting polymer, causing a shift towards lower or higher molecular weights, depending on the procedure used.

Piyasan Praserthdam’s [24] research on the effects of poisoning materials (e.g., Lewis bases) and impurity gases on ethylene polymerization provides additional context for understanding how epoxides affect polypropylene. The poisoning compounds investigated (methanol, acetone, and ethyl acetate) showed variations in the deactivation power, and a decrease in the catalytic activity was observed due to the reduction in the number of active sites without affecting the degree of isotacticity. Similarly, in our study, the addition of epoxides caused a reduction in catalytic activity reflected in a decrease in MFI and Mw, suggesting that epoxides act as poisons by interfering with the active centers of the catalyst.

The findings of this study highlight the importance of controlling epoxide concentrations in polymerization processes to maintain the desired properties of polypropylene. The presence of epoxides, even in small quantities, can lead to accelerated thermal degradation and significantly alter the polymer’s MFI and Mw, affecting its performance in end applications. These results underscore the need to purify feed streams of recycled monomers and strictly control impurities during polymer synthesis to ensure the quality and consistency of the final product.

## 5. Conclusions

This study has demonstrated through the application of advanced techniques such as TGA and FTIR, as well as correlation analysis between variables, that inhibitors such as ethylene oxide, propylene, 1,2-butene, and 1,2-pentene have a significant impact on the catalytic activity of the Ziegler–Natta, system. These inhibitors increase the system’s stability by forming coordination complexes with the catalyst, but at the same time, they substantially alter the average molecular weight of the resulting polymers. It is concluded that even minimum concentrations from 0.00063 ppm of these inhibitors drastically affect the catalytic efficiency, resulting in notable modifications in critical properties such as the melt flow index (MFI), the average molecular weight (Mw) and the flexural modulus (PSI) of polypropylene. These alterations make the resulting polymers inappropriate for specific applications requiring consistent mechanical and thermal properties. However, it is crucial to continue research to comprehensively understand the inhibition mechanisms of these additional substances and their effects on industrial polymerization.

## Figures and Tables

**Table 1:** Concentration of the inhibitors and nomenclature of the samples analyzed.

Inhibitor	Sample Nomenclature	Injected Concentrations (ppm)
Propylene oxide	M0	0
	M1	0.00063
	M2	0.00116
	M3	0.00226
	M4	0.0053
	M5	0.01
	M6	0.015
	M7	0.021
Ethylene oxide	M8	0
	M9	0.0666
	M10	0.1333
	M11	0.2733
	M12	0.5766
	M13	0.6566
	M14	0.6966
	M15	0.7433
1,2-butene oxide	M16	0
	M17	0.00012
	M18	0.00012
	M19	0.00313
	M20	0.01333
	M21	0.025
	M22	0.05266
1,2-pentene oxide	M24	0
	M25	0.00143
	M26	0.052
	M27	0.075
	M28	0.124
	M29	0.27266
	M30	0.662
	M31	1.24533

**Table 2:** Frequencies (cm^−1^) of characteristic bonds present in the catalyst and the oxides.

Bonds	ZN-E-P	ZN-OE	ZN-OP	ZN-O1,2B	ZN-O1,2P
Ti-O	--------	430	440	475	475
Ti-Cl	618–555	493	725	591	599
Cl-Mg	1510	1456	1510	1625	1634
Cl-Ti-O-CH_2_	--------	957	1037	1028	1037
-CH_3_	2990–2972	--------	3044	2981	3106–2990
-CH_2_	1510–1474	1456–1260	1510–1438	1501–1456	1501–1456

**Table 3:** Symbolic representation of the reactions of the process.

Reagents		Products
TiCl_4_ + C_3_H_7_O	→	C_2_H_4_OTiCl_4_
TiCl_4_ + C_3_H_6_O	→	C_3_H_6_OTiCl_4_
TiCl_4_ + C_4_H_8_O	→	C_4_H_8_OTiCl_4_
TiCl_4_ + C_4_H_8_O	→	C_5_H_10_OTiCl_4_

## Data Availability

Data are contained within the article.
